# (*E*)-1-(4-Bromo­phen­yl)but-2-en-1-one

**DOI:** 10.1107/S2414314625004158

**Published:** 2025-05-13

**Authors:** Marcel Sonneck, Anke Spannenberg, Sebastian Wohlrab, Tim Peppel

**Affiliations:** a Leibniz-Institut für Katalyse e V Albert-Einstein-Str 29a 18059 Rostock Germany; Vienna University of Technology, Austria

**Keywords:** crystal structure, unsaturated compound, carbonyl side chain, stacking inter­actions

## Abstract

The title compound, C_10_H_9_BrO, consists of a *para*-substituted bromo­phenyl core and an unsaturated carbonyl side chain. The angle between the plane through the carbon atoms of the aryl ring and the plane through the carbon atoms of the unsaturated side chain is 29.12 (16)°. The cohesion in the crystal is ensured by π–π stacking and C—H⋯O inter­actions.

## Abstract 2

The title compound consists of a *para*-substituted bromo­phenyl core and an unsaturated carbonyl side chain.

## Graphical abstract

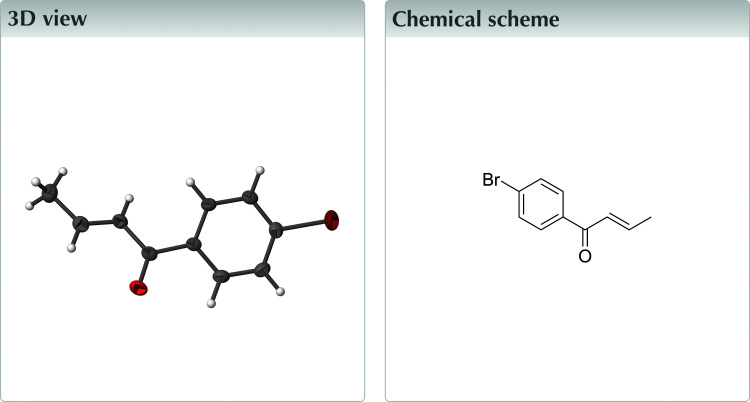

## Structure description

The structures of α,β-unsaturated carbonyl compounds are a common motif in a variety of natural products or bulk chemicals. These compounds are versatile synthetic inter­mediates for multiple organic transformation reactions, such as Michael addition, Diels–Alder reaction or Heck reaction (Ponec, 1997 ▸; Engel & Dudley, 2009 ▸; Desimoni *et al.*, 2018 ▸). The title compound, C_10_H_9_BrO, was received in low yield in high purity in a Friedel–Crafts acyl­ation. It can be designated as a suitable building block in the ongoing efforts to synthesize feasible new ligands for Cu-based complexes (Sonneck *et al.*, 2015 ▸, 2016 ▸).

**Figure 1:**
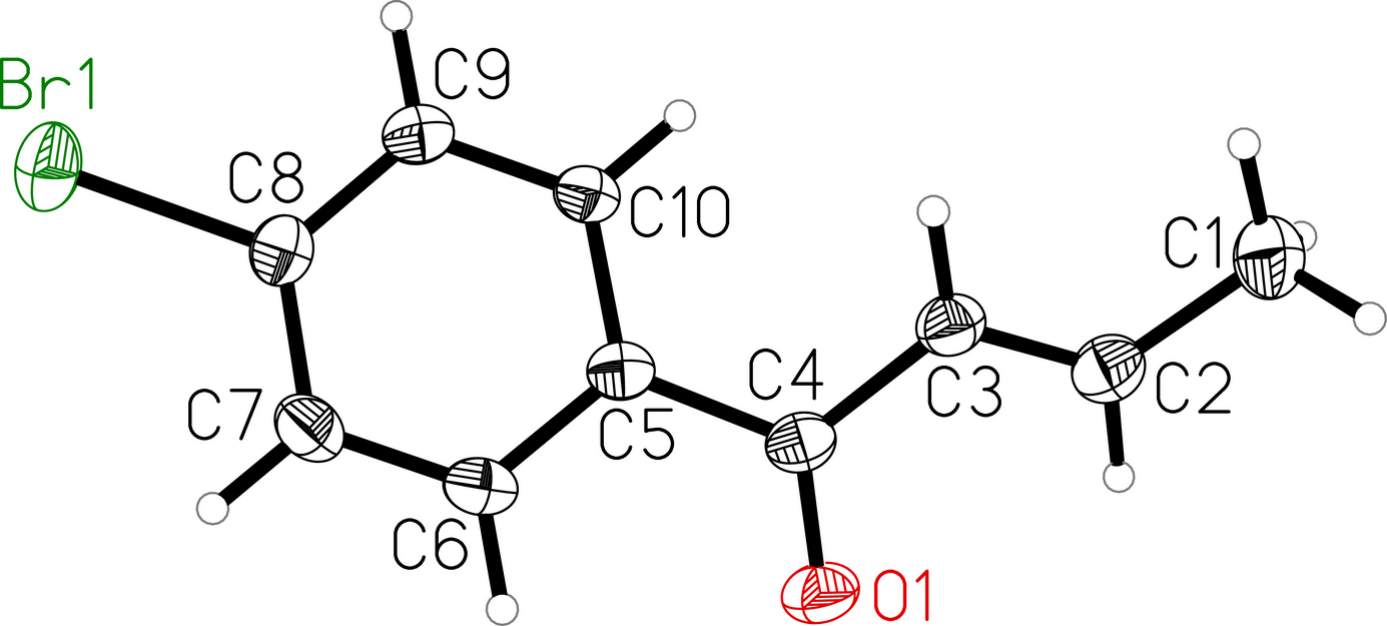
Mol­ecular structure of the title compound with atom labeling and displacement ellipsoids drawn at the 50% probability level.

**Figure 2:**
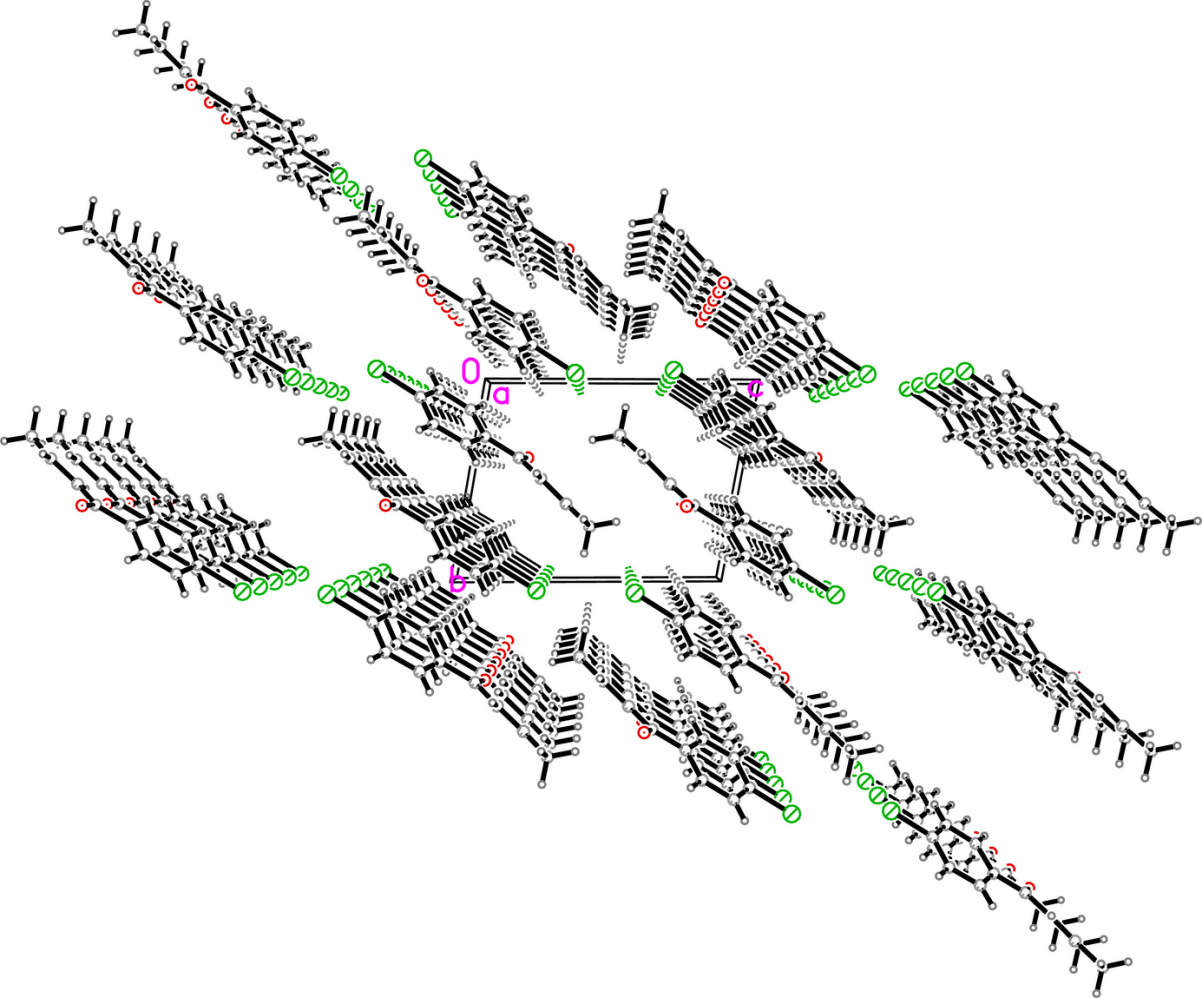
Packing diagram (ball-and-stick representation) for the title compound in a view along [100].

The mol­ecular structure of the title compound consists of a *para*-substituted bromo­phenyl core and an unsaturated carbonyl side chain (Fig. 1 ▸). The angle between the plane defined by the aryl ring (C5–C10) and the plane through the carbon atoms of the unsaturated side chain (C1–C4) is 29.12 (16)°. Carbonyl oxygen atom O1 is 0.246 (4) Å out of the latter plane. In the crystal, weak π–π stacking inter­actions between adjacent mol­ecules are observed, with a centroid(C5–C10)-to-centroid(C5–C10)’ distance of 3.724 (1) Å [ring slippage = 1.31 Å; symmetry code: (’) 1 − *x*, 2 − *y*, 2 − *z*]. Additionally, weak inter­molecular C—H⋯O inter­actions are present in the crystal packing (Table 1 ▸, Fig. 2 ▸). All bond lengths and angles are in expected ranges and the C=O bond length is 1.2278 (17) Å.

## Synthesis and crystallization

The title compound, C_10_H_9_BrO, was obtained as colorless crystals in low yield from the Friedel–Crafts acyl­ation of bromo­benzene and crotonyl chloride in CS_2_. AlCl_3_ (61.2 g, 459.2 mmol, 1.20 eq.) was added to a stirred solution of bromo­benzene (81.1 g, 516.6 mmol, 1.35 eq.) in 150 ml of CS_2_ at room temperature. Crotonyl chloride (40.0 g, 382.7 mmol, 1.00 eq.) was added dropwise to the thoroughly stirred suspension and afterwards the solution was heated under reflux for 24 h. The resulting red solution was poured onto a mixture of ice and concentrated hydro­chloric acid (500 g: 50 g) and extracted 3× with 150 ml portions of ethyl acetate. The volume of the combined organic phases was reduced to 150 ml and extracted twice with 100 ml portions of brine. The organic phase was dried with Na_2_SO_4_ and the solvent was removed completely under diminished pressure. The resulting raw product was distilled under reduced pressure to give an orange-colored distillate. After storing the distillate for several days at 243 K, colorless single crystals of the product were obtained in low yield (9.5 g, 11%).

Analytical data for C_10_H_9_BrO: mp. 323 K, elemental analysis % (calculated): C 53.40 (53.36), H 3.95 (4.03); Br 35.42 (35.50). ^1^H NMR (300 MHz, MeOD): δ (p.p.m.) = 8.75–8.67 (*m*, 2H, ArH); 8.56–8.48 (*m*, 2H, ArH); 8.01–7.81 (*m*, 2H); 2.87 (*d*, ^3^*J* = 6.0 Hz, 3H, –Me); ^13^C NMR (75 MHz, MeOD): δ (p.p.m.) = 191.15 (CO); 147.25 (CH); 137.84 (C); 132.98, 132.98, 141.29, 131.29 (CH), 128.78 (C), 127.97 (CH), 18.67 (CH_3_).

## Refinement

Crystal data, data collection and structure refinement details are summarized in Table 2 ▸.

## Supplementary Material

Crystal structure: contains datablock(s) I. DOI: 10.1107/S2414314625004158/wm4228sup1.cif

Structure factors: contains datablock(s) I. DOI: 10.1107/S2414314625004158/wm4228Isup2.hkl

Supporting information file. DOI: 10.1107/S2414314625004158/wm4228Isup3.cml

CCDC reference: 1495201

Additional supporting information:  crystallographic information; 3D view; checkCIF report

## Figures and Tables

**Table 1:** Hydrogen-bond geometry (Å, °)

*D*—H⋯*A*	*D*—H	H⋯*A*	*D*⋯*A*	*D*—H⋯*A*
C3—H3⋯O1^i^	0.95	2.56	3.5094 (18)	177
C1—H1*C*⋯O1^ii^	0.98	2.58	3.555 (2)	172

Symmetry codes: (i) 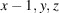; (ii) 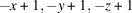.

**Table 2:** Experimental details

Crystal data	
Chemical formula	C_10_H_9_BrO
*M*_r_	225.08
Crystal system, space group	Triclinic, *P* 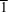
Temperature (K)	150
*a*, *b*, *c* (Å)	5.5734 (9), 8.1618 (13), 10.6194 (16)
α, β, γ (°)	98.577 (2), 96.441 (2), 102.546 (2)
*V* (Å^3^)	461.00 (13)
*Z*	2
Radiation type	Mo *K*α
μ (mm^−1^)	4.41
Crystal size (mm)	0.36 × 0.23 × 0.04

Data collection	
Diffractometer	Bruker APEXII CCD
Absorption correction	Multi-scan (*SADABS*; Krause *et al.*, 2015 ▸)
*T*_min_, *T*_max_	0.63, 0.85
No. of measured, independent and observed [*I* > 2σ(*I*)] reflections	12194, 2232, 2077
*R*_int_	0.027
(sin θ/λ)_max_ (Å^−1^)	0.661

Refinement	
*R*[*F*^2^ > 2σ(*F*^2^)], *wR*(*F*^2^), *S*	0.019, 0.051, 1.05
No. of reflections	2232
No. of parameters	110
H-atom treatment	H-atom parameters constrained
Δρ_max_, Δρ_min_ (e Å^−3^)	0.32, −0.27

Computer programs: *APEX2* and *SAINT* (Bruker, 2014 ▸), *SHELXS97* (Sheldrick, 2008 ▸), *SHELXL* (Sheldrick, 2015 ▸), *XP* in *SHELXTL* (Sheldrick, 2008 ▸) and *publCIF* (Westrip, 2010 ▸).

## full crystallographic data

### (E)-1-(4-Bromophenyl)but-2-en-1-one Crystal data

**Table:**

C_10_H_9_BrO	*Z* = 2
*M**_r_* = 225.08	*F*(000) = 224
Triclinic, *P*1	*D*_x_ = 1.622 Mg m^−^^3^
*a* = 5.5734 (9) Å	Mo *K*α radiation, λ = 0.71073 Å
*b* = 8.1618 (13) Å	Cell parameters from 7348 reflections
*c* = 10.6194 (16) Å	θ = 2.6–28.6°
α = 98.577 (2)°	µ = 4.41 mm^−^^1^
β = 96.441 (2)°	*T* = 150 K
γ = 102.546 (2)°	Plate, colourless
*V* = 461.00 (13) Å^3^	0.36 × 0.23 × 0.04 mm

### (E)-1-(4-Bromophenyl)but-2-en-1-one Data collection

**Table:**

Bruker APEXII CCD diffractometer	2232 independent reflections
Radiation source: fine-focus sealed tube	2077 reflections with *I* > 2σ(*I*)
Detector resolution: 8.3333 pixels mm^-1^	*R*_int_ = 0.027
φ and ω scans	θ_max_ = 28.0°, θ_min_ = 2.0°
Absorption correction: multi-scan (SADABS; Krause *et al.*, 2015)	*h* = −7→7
*T*_min_ = 0.63, *T*_max_ = 0.85	*k* = −10→10
12194 measured reflections	*l* = −14→14

### (E)-1-(4-Bromophenyl)but-2-en-1-one Refinement

**Table:**

Refinement on *F*^2^	0 restraints
Least-squares matrix: full	Hydrogen site location: inferred from neighbouring sites
*R*[*F*^2^ > 2σ(*F*^2^)] = 0.019	H-atom parameters constrained
*wR*(*F*^2^) = 0.051	*w* = 1/[σ^2^(*F*_o_^2^) + (0.0243*P*)^2^ + 0.1552*P*] where *P* = (*F*_o_^2^ + 2*F*_c_^2^)/3
*S* = 1.05	(Δ/σ)_max_ = 0.002
2232 reflections	Δρ_max_ = 0.32 e Å^−^^3^
110 parameters	Δρ_min_ = −0.27 e Å^−^^3^

### (E)-1-(4-Bromophenyl)but-2-en-1-one Special details

**Table:**

Geometry. All esds (except the esd in the dihedral angle between two l.s. planes) are estimated using the full covariance matrix. The cell esds are taken into account individually in the estimation of esds in distances, angles and torsion angles; correlations between esds in cell parameters are only used when they are defined by crystal symmetry. An approximate (isotropic) treatment of cell esds is used for estimating esds involving l.s. planes.

### (E)-1-(4-Bromophenyl)but-2-en-1-one Fractional atomic coordinates and isotropic or equivalent isotropic displacement parameters (Å^2^)

**Table:**

	*x*	*y*	*z*	*U*_iso_*/*U*_eq_	
Br1	0.46488 (3)	1.00661 (2)	1.33348 (2)	0.03862 (7)	
C1	0.1030 (3)	0.2949 (2)	0.52287 (17)	0.0397 (4)	
H1A	−0.047264	0.328231	0.545930	0.060*	
H1B	0.086027	0.173332	0.524923	0.060*	
H1C	0.125813	0.316143	0.436060	0.060*	
C2	0.3226 (3)	0.39689 (19)	0.61669 (15)	0.0308 (3)	
H2	0.480779	0.377596	0.603523	0.037*	
C3	0.3146 (3)	0.51246 (19)	0.71713 (14)	0.0274 (3)	
H3	0.158932	0.534668	0.732273	0.033*	
C4	0.5417 (3)	0.60755 (18)	0.80609 (14)	0.0253 (3)	
C5	0.5149 (2)	0.70343 (17)	0.93299 (13)	0.0230 (3)	
C6	0.7179 (3)	0.82873 (19)	1.00146 (15)	0.0284 (3)	
H6	0.867758	0.852042	0.965647	0.034*	
C7	0.7057 (3)	0.91961 (19)	1.12013 (15)	0.0304 (3)	
H7	0.843908	1.006229	1.165242	0.037*	
C8	0.4872 (3)	0.88158 (18)	1.17190 (13)	0.0260 (3)	
C9	0.2838 (3)	0.75609 (19)	1.10791 (14)	0.0276 (3)	
H9	0.136975	0.729938	1.145979	0.033*	
C10	0.2970 (2)	0.66900 (18)	0.98747 (14)	0.0257 (3)	
H10	0.156462	0.585051	0.941560	0.031*	
O1	0.75060 (19)	0.60866 (15)	0.77924 (11)	0.0348 (2)	

### (E)-1-(4-Bromophenyl)but-2-en-1-one Atomic displacement parameters (Å^2^)

**Table:**

	*U*^11^	*U*^22^	*U*^33^	*U*^12^	*U*^13^	*U*^23^
Br1	0.05075 (11)	0.03740 (10)	0.02638 (9)	0.01081 (7)	0.00651 (7)	0.00052 (6)
C1	0.0426 (9)	0.0367 (9)	0.0352 (8)	0.0058 (7)	0.0048 (7)	−0.0022 (7)
C2	0.0319 (7)	0.0300 (7)	0.0329 (8)	0.0098 (6)	0.0087 (6)	0.0068 (6)
C3	0.0246 (7)	0.0318 (7)	0.0276 (7)	0.0090 (6)	0.0066 (5)	0.0061 (6)
C4	0.0243 (6)	0.0268 (7)	0.0282 (7)	0.0091 (5)	0.0071 (5)	0.0086 (5)
C5	0.0212 (6)	0.0244 (6)	0.0259 (6)	0.0074 (5)	0.0045 (5)	0.0085 (5)
C6	0.0207 (6)	0.0317 (7)	0.0326 (7)	0.0035 (5)	0.0053 (5)	0.0083 (6)
C7	0.0256 (7)	0.0310 (7)	0.0302 (7)	0.0001 (6)	−0.0005 (5)	0.0044 (6)
C8	0.0310 (7)	0.0265 (7)	0.0219 (6)	0.0093 (6)	0.0032 (5)	0.0059 (5)
C9	0.0249 (6)	0.0307 (7)	0.0291 (7)	0.0063 (6)	0.0082 (5)	0.0083 (6)
C10	0.0210 (6)	0.0261 (7)	0.0287 (7)	0.0027 (5)	0.0049 (5)	0.0047 (5)
O1	0.0239 (5)	0.0467 (7)	0.0360 (6)	0.0114 (5)	0.0098 (4)	0.0063 (5)

### (E)-1-(4-Bromophenyl)but-2-en-1-one Geometric parameters (Å, º)

**Table:**

Br1—C8	1.8925 (14)	C5—C6	1.395 (2)
C1—C2	1.490 (2)	C5—C10	1.3951 (18)
C1—H1A	0.9800	C6—C7	1.380 (2)
C1—H1B	0.9800	C6—H6	0.9500
C1—H1C	0.9800	C7—C8	1.385 (2)
C2—C3	1.326 (2)	C7—H7	0.9500
C2—H2	0.9500	C8—C9	1.382 (2)
C3—C4	1.478 (2)	C9—C10	1.385 (2)
C3—H3	0.9500	C9—H9	0.9500
C4—O1	1.2278 (17)	C10—H10	0.9500
C4—C5	1.4940 (19)		
			
C2—C1—H1A	109.5	C10—C5—C4	122.52 (13)
C2—C1—H1B	109.5	C7—C6—C5	121.43 (13)
H1A—C1—H1B	109.5	C7—C6—H6	119.3
C2—C1—H1C	109.5	C5—C6—H6	119.3
H1A—C1—H1C	109.5	C6—C7—C8	118.46 (14)
H1B—C1—H1C	109.5	C6—C7—H7	120.8
C3—C2—C1	125.10 (14)	C8—C7—H7	120.8
C3—C2—H2	117.4	C9—C8—C7	121.73 (13)
C1—C2—H2	117.4	C9—C8—Br1	119.11 (11)
C2—C3—C4	121.66 (13)	C7—C8—Br1	119.16 (11)
C2—C3—H3	119.2	C8—C9—C10	119.06 (13)
C4—C3—H3	119.2	C8—C9—H9	120.5
O1—C4—C3	122.00 (13)	C10—C9—H9	120.5
O1—C4—C5	119.30 (13)	C9—C10—C5	120.62 (13)
C3—C4—C5	118.69 (12)	C9—C10—H10	119.7
C6—C5—C10	118.67 (13)	C5—C10—H10	119.7
C6—C5—C4	118.78 (12)		
			
C1—C2—C3—C4	179.68 (14)	C5—C6—C7—C8	1.2 (2)
C2—C3—C4—O1	13.9 (2)	C6—C7—C8—C9	0.0 (2)
C2—C3—C4—C5	−165.45 (14)	C6—C7—C8—Br1	−179.19 (11)
O1—C4—C5—C6	17.9 (2)	C7—C8—C9—C10	−1.6 (2)
C3—C4—C5—C6	−162.71 (13)	Br1—C8—C9—C10	177.61 (10)
O1—C4—C5—C10	−160.10 (14)	C8—C9—C10—C5	2.0 (2)
C3—C4—C5—C10	19.2 (2)	C6—C5—C10—C9	−0.8 (2)
C10—C5—C6—C7	−0.8 (2)	C4—C5—C10—C9	177.24 (13)
C4—C5—C6—C7	−178.94 (13)		

### (E)-1-(4-Bromophenyl)but-2-en-1-one Hydrogen-bond geometry (Å, º)

**Table:**

*D*—H···*A*	*D*—H	H···*A*	*D*···*A*	*D*—H···*A*
C3—H3···O1^i^	0.95	2.56	3.5094 (18)	177
C1—H1*C*···O1^ii^	0.98	2.58	3.555 (2)	172

Symmetry codes: (i) *x*−1, *y*, *z*; (ii) −*x*+1, −*y*+1, −*z*+1.
